# High neutrophil to lymphocyte ratio with type 2 diabetes mellitus predicts poor prognosis in patients undergoing percutaneous coronary intervention: a large-scale cohort study

**DOI:** 10.1186/s12933-022-01583-9

**Published:** 2022-08-13

**Authors:** Jining He, Xiaohui Bian, Chenxi Song, Rui Zhang, Sheng Yuan, Dong Yin, Kefei Dou

**Affiliations:** 1 State Key Laboratory of Cardiovascular Disease, Beijing, China; 2 Cardiometabolic Medicine Center, Fuwai Hospital, National Center for Cardiovascular Diseases, Chinese Academy of Medical Sciences and Peking Union Medical College, 167A Beilishi Road, Xi Cheng District, Beijing, 100037 China; 3 Department of Cardiology, Fuwai Hospital, National Center for Cardiovascular Diseases, Chinese Academy of Medical Sciences and Peking Union Medical College, Beijing, China

**Keywords:** Neutrophil‑to‑lymphocyte ratio, Diabetes, Prognosis, Coronary artery disease, Percutaneous coronary intervention

## Abstract

**Background:**

Inflammation plays a crucial role in the pathogenesis and progression of coronary artery disease (CAD). The neutrophil to lymphocyte ratio (NLR) is a novel inflammatory biomarker and its association with clinical outcomes in CAD patients with different glycemic metabolism after percutaneous coronary intervention (PCI) remains undetermined. Therefore, this study aimed to investigate the effect of NLR on the prognosis of patients undergoing PCI with or without type 2 diabetes mellitus (T2DM).

**Methods:**

We consecutively enrolled 8,835 patients with CAD hospitalized for PCI at Fuwai hospital. NLR was calculated using the following formula: neutrophil (*10^9^/L)/lymphocyte (*10^9^/L). According to optimal cut-off value, study patients were categorized as higher level of NLR (NLR-H) and lower level of NLR (NLR-L) and were further stratified as NLR-H with T2DM and non-T2DM, and NLR-L with T2DM and non-T2DM. The primary endpoint was major adverse cardiovascular and cerebrovascular events (MACCEs), defined as all-cause mortality, myocardial infarction (MI), stroke and target vessel revascularization.

**Results:**

A total of 674 (7.6%) MACCEs were recorded during a median follow-up of 2.4 years. The optimal cut-off value of NLR was 2.85 determined by the surv_cutpoint function. Compared to those in the NLR-H/T2DM groups, patients in the NLR-L/non-T2DM, NLR-H/non-T2DM and NLR-L/T2DM groups were at significantly lower risk of 2-year MACCEs [adjusted hazard ratio (HR): 0.67, 95% confidence interval (CI): 0.52 to 0.87, P = 0.003; adjusted HR: 0.62, 95%CI: 0.45 to 0.85, P = 0.003; adjusted HR: 0.77, 95%CI: 0.61 to 0.97, P = 0.025; respectively]. Remarkably, patients in the NLR-L/non-T2DM group also had significantly lower risk of a composite of all-cause mortality and MI than those in the NLR-H/T2DM group (adjusted HR: 0.57, 95%CI: 0.35 to 0.93, P = 0.024). Multivariable Cox proportional hazards model also indicated the highest risk of MACCEs in diabetic patients with higher level of NLR than others (P for trend = 0.009). Additionally, subgroup analysis indicated consistent impact of NLR on MACCEs across different subgroups.

**Conclusions:**

Presence of T2DM with elevated NLR is associated with worse clinical outcomes in CAD patients undergoing PCI. Categorization of patients with elevated NLR and T2DM could provide valuable information for risk stratification of CAD patients.

**Supplementary Information:**

The online version contains supplementary material available at 10.1186/s12933-022-01583-9.

## Introduction

The nature history of coronary artery disease (CAD) is complex and includes multiple clinical stages. Inflammation plays a crucial role in the pathogenesis and progression of CAD [1]. Circulating white blood cell (WBC) count was an established biomarker for inflammation [2]. It plays a leading role in the processes of the vascular wall degeneration and being involved in the acceleration of atherosclerosis and in the destabilization and rupture of plaque, leading to thrombotic events [3, 4]. Previous studies have reported elevated WBC counts could independently predicted mortality and major adverse events for patients with acute coronary syndrome (ACS) [5, 6]. Subsequently, the impact of leucocyte subtypes on prognosis in CAD patients were widely explored. Neutrophils are of importance to stabilize the atherosclerotic plaques. Previous systematic review on over 34 thousand patients demonstrated that neutrophil was an independent predictor for cardiovascular events when analyzed simultaneously with other inflammatory biomarkers such as WBC and C-reactive protein (CRP), highlighting its potential role of risk stratification in patients with ACS and/or cardiac revascularization [7]. Similarly, previous studies have reported the association between low lymphocyte count and poor prognosis in patients in different stages of CAD [8, 9].

As a newly emerged inflammatory biomarker, the neutrophil-to-lymphocyte ratio (NLR) integrates the information of the leukocyte differentials into one variable and provides a better prognostic value than each parameter separately [10]. Previous studies have shown that an increased NLR was intensively linked to the progression of coronary atherosclerosis [11]. Higher ratios were in association with worse cardiovascular risk profile, and complexity and severity of CAD evaluated by the number of diseased vessels, the SYNergy between percutaneous coronary intervention with TAXus and cardiac surgery (SYNTAX) score and the Gensini score [12, 13]. Consequently, patients with higher ratios could encounter higher risk for all-cause mortality and cardiovascular events [13].

Inflammation is regarded as the common antecedent of atherosclerosis and type 2 diabetes mellitus (T2DM) [14]. T2DM, as a well-established risk factor for CAD, had been previously demonstrated to be closely related to greater atherosclerotic plaque burden and increased risk of poor clinical outcomes [15, 16]. There is, to date, a scarcity of literature investigating the relationship between NLR and clinical outcomes in CAD patients with different glycemic metabolism status after percutaneous coronary intervention (PCI). Therefore, this large, prospective cohort study was conducted to investigate the prognosis of diabetic and non-diabetic patients undergoing PCI with different level of NLR.

## Methods

### Study design

**Fig. 1:**
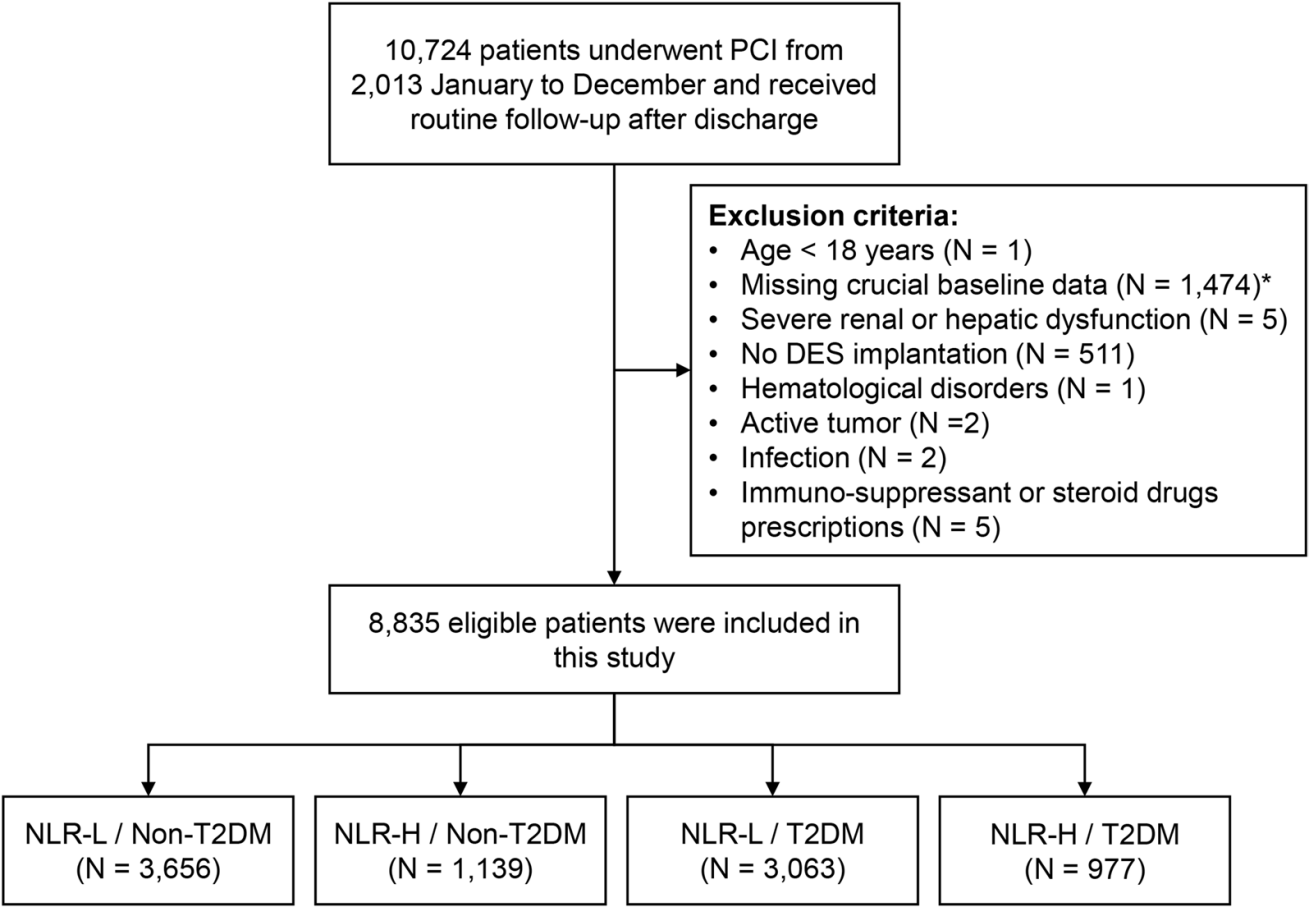
Study flowchart. *A total of 708 patients with missing neutrophil or lymphocyte count and 845 patients with missing FBG or HbA1c levels were excluded. PCI, percutaneous coronary intervention; DES, drug-eluting stent; other abbreviations as in Table 1

The present study was a prospective, observational cohort study at Fuwai hospital, Chinese Academy of Medical Sciences [17]. From January 2013 to December 2013, a total of 10,724 patients who underwent PCI at Fuwai hospital were consecutively screened. Patients were eligible according to the following: (1) CAD patients treated with drug-eluting stent (DES) implantation; and (2) aged over 18 years. Patients with missing crucial baseline laboratory data and/or other exclusion criteria were excluded (Fig. 1). Ultimately, a total of 8835 patients were included in this study and divided into the NLR-H/T2DM (n = 977), NLR-L/T2DM (n = 3063), NLR-H/Non-T2DM (n = 1139) and NLR-L/Non-T2DM (n = 3656) groups, according to the optimal cut-off value of NLR and distinct glycemic metabolism status.

The clinical status was evaluated at 1, 6, and 12 months and yearly thereafter by outpatient visits or telephone interview. The primary endpoint was major adverse cardiovascular and cerebrovascular events (MACCEs) during follow-up, defined as a composite of all-cause mortality, myocardial infarction (MI), stroke, and target vessel revascularization (TVR). The secondary outcomes were a composite of all-cause mortality and MI, and individual components of MACCEs. All-cause mortality was defined as death from any cause, either cardiac or noncardiac. MI was determined based on clinical and laboratory parameters, according to the third universal definition of MI [18]. TVR was defined as any repeat revascularization of any segment of the target vessel. Stroke was defined as neurological deficits, either ischemic or hemorrhagic, confirmed by a neurologist based on imaging findings [19]. All events were adjudicated by two independent experienced cardiologists, unaware of this study. Conflicts were resolved by consulting a third experienced cardiologist.

The study process was in accordance with the Declaration of Helsinki and was approved by the Institutional Review Board of Fuwai hospital. All subjects provided informed written consent for long-term follow-up before intervention.

### Procedures

PCI was performed by experienced interventionalists in line with standard techniques. The choice of devices, adjunctive examinations (i.e., intravascular ultrasound and optical coherence tomography), and detailed strategies were left to the discretion of operators. Before the scheduled PCI, aspirin (300 mg) and a P2Y12 inhibitor (clopidogrel 300–600 mg or ticagrelor 180 mg) were administered to all patients unfractionated heparin or bivalirudin were used to achieve procedural anticoagulation. After the catheterization, aspirin 100 mg/day was prescribed indefinitely and clopidogrel 75 mg/day typically for 12 months. Data were entered in a dedicated database by independent research personnel [20, 21].

### Data collection and definitions

Demographic information and clinical data were prospectively gathered for all patients. Demographic information comprised age, sex, weight, height, comorbidities, smoking status, and previous MI or revascularization history (PCI or coronary artery bypass grafting [CABG]). Clinical data included the main diagnosis on admission, physical, imaging, and laboratory examination, and medication regimen at discharge.

T2DM was determined by fasting blood glucose (FBG) ≥ 7.0 mmol/L (126 mg/dL), or hemoglobin A1c (HbA1c) levels ≥ 6.5%, or 2-h blood glucose of oral glucose tolerance test ≥ 11.1 mmol/L (200 mg/dL), or previous definite diagnosis of T2DM with hypoglycemic drugs treatment [22]. NLR was calculated using the following formula: $\frac{\mathrm{plasma neutrophil count }\left(*{10}^{9}/\mathrm{L}\right)}{\mathrm{ plasma lymphocyte count }(*{10}^{9}/\mathrm{L})}$ [23]. Hypertension was defined as newly confirmation more than twice on different days by systolic blood pressure ≥ 140 mmHg and/or diastolic blood pressure ≥ 90 mmHg during the baseline hospitalization or known hypertension with antihypertensive medication. Patients with fasting total cholesterol (TC) ≥ 5.2 mmol/L, low-density lipoprotein cholesterol (LDL-C) ≥ 3.4 mmol/L, high-density lipoprotein cholesterol (HDL-C) < 1.0 mmol/L, triglyceride (TG) ≥ 1.7 mmol/L, and/or receiving lipid-lowering medications were diagnosed as dyslipidemia [24]. Renal dysfunction was recorded when estimated glomerular filtration rate (eGFR) less than 90 mL/(min*1.73m^2^). Body mass index (BMI) was calculated by weight (kg) divided by the square of height (m).

After the PCI procedures, the characteristics of the coronary disease were interpreted and recorded by two independent blinded intervention specialists and disagreement was resolved by consensus. Based on the coronary angiography, left main (LM) disease was determined by ≥ 50% stenosis in LM coronary artery and three-vessel disease was defined as ≥ 50% in all three coronary epicardial arteries (i.e., the left anterior descending artery, left circumflex artery, and right coronary artery). Chronic total occlusion (CTO) was defined as complete occlusion of a native coronary artery for more than 3 months with thrombolysis in myocardial infarction (TIMI) flow grade of 0. The SYNTAX score was estimated using an online calculator (http://www.syntaxscore.com/) to assess the coronary lesion complexity by a dedicated blinded research group.

### Laboratory tests and echocardiography

Fasting blood samples were routinely drawn from each patient on the day of admission and all of them were stored in − 80 °C refrigerators until test. Complete blood count including WBC, neutrophil, lymphocyte, and other parameters was carried out using an automatic blood cell analyzer (XT-1800i; Sysmex Corporation) [23]. The HbA1c levels were measured by Tosoh Automated Glycohemoglobin Analyzer (HLC-723G8, Tokyo, Japan) [20]. Other laboratory parameters, including lipid profiles (TG, TC, HDL-C and LDL-C), eGFR, creatinine, high-sensitivity CRP (hsCRP) were examined at the core laboratory in Fuwai hospital, according to the standard operating procedures [20]. The eGFR was calculated using the Chinese-modified MDRD (Modification of Diet in Renal Disease) equation [25]. Based on modified Simpson’s rule, left ventricular ejection fraction (LVEF) was estimated from two-dimensional echocardiography.

### Statistical analyses

Continuous variables were described as mean with standard deviation and categorical variables were summarized as frequency with percentage. Comparison of continuous and categorical variables among different groups was analyzed by Student’s t-test or Mann–Whitney U test and Chi-square test or Fisher’s exact test, as appropriate.

Linear regression analysis was constructed to evaluate the association between NLR and glycemic or inflammatory parameters (FBG, HbA1c and hsCRP). In survival analysis, linearity assumption of NLR for the risk of MACCEs was depicted by restricted cubic splines and examined by likelihood ratio test. The risks of MACCEs in various groups were presented by Kaplan–Meier survival curves and compared by log-rank test. Proportional hazards assumption was determined by Schoenfeld residuals. The combined impact of NLR and glycemic metabolism status on adverse clinical events were estimated by univariable and multivariable Cox regression analyses using the NLR-H/T2DM group as reference. Hazard ratios (HRs) and 95% confidence interval (CI) were presented. In the multivariable Cox analysis, NLR, age, male sex, hypertension, dyslipidemia, smoking history, previous MI, previous PCI, previous stroke, previous peripheral artery disease (PAD), ACS, HbA1c, TG, LDL-C, hsCRP, eGFR, LVEF, dual antiplatelet therapy (DAPT), β blocker, LM/three-vessel disease, CTO, moderate to severe calcification, number of treated vessels, number of stents, IABP use and SYNTAX score were included due to their statistical significance in univariable analysis or clinical importance. We performed collinearity analysis to explore the collinearity of variables included in the multivariable model. Moreover, we conducted subgroup analyses of the risk of MACCEs among six different subsets and exhibited as the forest plot. A two-tailed *P* value < 0.05 was considered statistical significance. Statistical analyses were performed using SPSS version 25.0 (IBM Corporation, Armonk, NY) and RStudio software (version 2021.09.0; http://www.rstudio.org/).

## Results

### Baseline characteristics

Generally, 8835 consecutive CAD patients (58.38 ± 10.19 years, 77.0% male) who received PCI with DES implantation were included in this study. During a median follow-up of 2.4 years (interquartile range: 2.2–2.6 years), 104 (1.2%) all-cause mortality, 97 (1.1%) MI, 139 (1.6%) stroke, 417 (4.7%) TVR, 174 (2.0%) a composite of all-cause mortality and MI, and 674 (7.6%) MACCEs were recorded.

Table 1 lists the baseline characteristics of patients grouped by the occurrence of MACCEs. Patients with any component of MACCEs tended to have an advanced age, with a larger burden of concomitant diseases, including T2DM, hypertension, previous MI, and previous stroke compared with those in non-MACCE group. Besides, higher neutrophils count, FBG, HbA1c, hsCRP, and lower LVEF, eGFR were observed in patients with poor prognosis. As for the angiographic characteristics, patients in the MACCE group were more likely to have LM/three-vessel disease, CTO lesions, moderate to severe calcification. Consequently, significantly higher SYNTAX score, number of treated vessels and number of stents were noted in patients with adverse prognosis.

**Table 1:** Baseline characteristics for patients with or without MACCEs

Variables	Overall (N = 8835)	Non-MACCE (N = 8161)	MACCE (N = 674)	*P* value
Four subgroups				< 0.001
NLR-L/Non-T2DM	3656 (41.4)	3419 (41.9)	237 (35.2)	
NLR-H/Non-T2DM	1139 (12.9)	1066 (13.1)	73 (10.8)	
NLR-L/T2DM	3063 (34.7)	2811 (34.4)	252 (37.4)	
NLR-H/T2DM	977 (11.1)	865 (10.6)	112 (16.6)	
Baseline characteristics				
Age, years	58.38 ± 10.19	58.24 ± 10.16	60.08 ± 10.41	< 0.001
Male	6805 (77.0)	6286 (77.0)	519 (77.0)	1.000
BMI, kg/m^2^	25.92 ± 3.20	25.92 ± 3.21	25.92 ± 3.11	0.776
T2DM	4040 (45.7)	3676 (45.0)	364 (54.0)	< 0.001
Hypertension	5712 (64.7)	5247 (64.3)	465 (69.0)	0.016
Dyslipidemia	5912 (66.9)	5449 (66.8)	463 (68.7)	0.328
Smoking history	5055 (57.2)	4675 (57.3)	380 (56.4)	0.678
Previous MI	1678 (19.0)	1524 (18.7)	154 (22.8)	0.009
Previous PCI	2028 (23.0)	1848 (22.6)	180 (26.7)	0.018
Previous CABG	352 (4.0)	318 (3.9)	34 (5.0)	0.173
Previous stroke	927 (10.5)	829 (10.2)	98 (14.5)	< 0.001
Previous PAD	654 (7.4)	601 (7.4)	53 (7.9)	0.690
Clinical presentation				0.709
SAP	3619 (41.0)	3348 (41.0)	271 (40.2)	
ACS	5216 (59.0)	4813 (59.0)	403 (59.8)	
Laboratory tests				
Neutrophils, *10^9^/L	4.27 ± 1.57	4.25 ± 1.55	4.51 ± 1.76	< 0.001
Lymphocyte, *10^9^/L	1.95 ± 0.64	1.95 ± 0.64	1.97 ± 0.65	0.306
NLR	2.44 ± 1.57	2.43 ± 1.57	2.55 ± 1.54	0.051
FBG, mmol/L	6.28 ± 2.25	6.25 ± 2.20	6.67 ± 2.77	< 0.001
HbA1c, %	6.62 ± 1.25	6.61 ± 1.25	6.77 ± 1.24	< 0.001
TG, mmol/L	1.78 ± 1.08	1.78 ± 1.08	1.75 ± 1.03	0.420
TC, mmol/L	4.20 ± 1.06	4.20 ± 1.07	4.21 ± 1.02	0.702
HDL-C, mmol/L	1.04 ± 0.30	1.04 ± 0.30	1.05 ± 0.30	0.670
LDL-C, mmol/L	2.49 ± 0.90	2.49 ± 0.91	2.49 ± 0.87	0.825
hsCRP, mg/L	3.03 ± 3.60	3.00 ± 3.59	3.33 ± 3.71	0.002
Creatinine, μmol/L	75.25 ± 15.47	75.18 ± 15.43	75.98 ± 15.89	0.279
eGFR, mL/min/1.73 m^2^	102.84 ± 22.10	102.94 ± 21.96	101.53 ± 23.69	0.050
LVEF, %	63.08 ± 7.06	63.16 ± 7.01	62.15 ± 7.60	0.001
Medications				
DAPT	8636 (97.7)	7975 (97.7)	661 (98.1)	0.650
β-blocker	7955 (90.0)	7333 (89.9)	622 (92.3)	0.050
CCB	4353 (49.3)	3998 (49.0)	355 (52.7)	0.072
Statins	8484 (96.0)	7836 (96.0)	648 (96.1)	0.955
Nitrate	8648 (97.9)	7987 (97.9)	661 (98.1)	0.831
Coronary procedural data				
LM/three-vessel disease	3941 (44.6)	3582 (43.9)	359 (53.3)	< 0.001
Chronic total occlusion	726 (8.2)	656 (8.0)	70 (10.4)	0.039
Bifurcation lesions	1785 (20.2)	1661 (20.4)	124 (18.4)	0.244
Moderate to severe calcification	1560 (17.7)	1417 (17.4)	143 (21.2)	0.014
Number of treated vessels	1.43 ± 0.68	1.42 ± 0.67	1.52 ± 0.78	0.003
Number of stents	1.93 ± 1.06	1.92 ± 1.06	2.02 ± 1.04	0.003
IABP	84 (1.0)	64 (0.8)	20 (3.0)	< 0.001
SYNTAX score	11.91 ± 7.67	11.82 ± 7.61	13.01 ± 8.25	< 0.001

Values are mean ± standard deviation or n (%)

MACCE, major adverse cardiovascular and cerebrovascular events; NLR, neutrophil to lymphocyte ratio; T2DM, type 2 diabetes mellitus; BMI, body mass index; MI, myocardial infarction; PCI, percutaneous coronary intervention; CABG, coronary artery bypass grafting; PAD, peripheral artery disease; SAP, stable angina pectoris; ACS, acute coronary syndrome; FBG, fasting blood glucose; HbA1c, glycosylated hemoglobin A1c; TG, triglyceride; TC, total cholesterol; HDL-C, high-density lipoprotein cholesterol; LDL-C, low-density lipoprotein cholesterol; hsCRP, high-sensitivity C-reactive protein; eGFR, estimated glomerular filtration rate; LVEF, left ventricular ejection fraction; DAPT, dual antiplatelet therapy; CCB, calcium channel blocker; LM, left main; IABP, intra-aortic balloon pump; SYNTAX, synergy between PCI with taxus and cardiac surgery

### Baseline characteristics of patients in four groups

Upon the surv_cutpoint function of the R package survminer in the R programming language, the optimal cut-off value of NLR for the risk of MACCEs is 2.85. Consequently, baseline characteristics of four groups based on NLR levels and glycemic metabolism status were listed in Table 2.

**Table 2:** Baseline characteristics for diabetic or non-diabetic patients with different NLR levels

Variables	NLR-L/Non-T2DM (N = 3656)	NLR-H/Non-T2DM (N = 1139)	NLR-L/T2DM (N = 3063)	NLR-H/T2DM (N = 977)	*P* Value
Baseline characteristics					
Age, years	57.09 ± 10.29	58.77 ± 10.43	59.03 ± 9.71	60.74 ± 10.36	< 0.001
Male	2843 (77.8)	955 (83.8)	2234 (72.9)	773 (79.1)	< 0.001
BMI, kg/m^2^	25.70 ± 3.15	25.36 ± 3.29	26.37 ± 3.19	26.01 ± 3.13	< 0.001
Hypertension	2182 (59.7)	740 (65.0)	2074 (67.7)	716 (73.3)	< 0.001
Dyslipidemia	2325 (63.6)	709 (62.2)	2172 (70.9)	706 (72.3)	< 0.001
Smoking history	2125 (58.1)	678 (59.5)	1684 (55.0)	568 (58.1)	0.016
Previous MI	649 (17.8)	207 (18.2)	611 (19.9)	211 (21.6)	0.016
Previous PCI	741 (20.3)	220 (19.3)	773 (25.2)	294 (30.1)	< 0.001
Previous CABG	111 (3.0)	48 (4.2)	150 (4.9)	43 (4.4)	0.001
Previous stroke	312 (8.5)	113 (9.9)	372 (12.1)	130 (13.3)	< 0.001
Previous PAD	233 (6.4)	75 (6.6)	255 (8.3)	91 (9.3)	0.001
Clinical presentation					< 0.001
SAP	1478 (40.4)	430 (37.8)	1356 (44.3)	355 (36.3)	
ACS	2178 (59.6)	709 (62.2)	1707 (55.7)	622 (63.7)	
Laboratory tests					
Neutrophils, *10^9^/L	3.74 ± 1.11	5.33 ± 1.59	3.94 ± 1.14	6.02 ± 2.23	< 0.001
Lymphocyte, *10^9^/L	2.05 ± 0.57	1.42 ± 0.42	2.17 ± 0.65	1.48 ± 0.45	< 0.001
NLR	1.89 ± 0.51	3.95 ± 1.70	1.89 ± 0.50	4.47 ± 2.87	< 0.001
FBG, mmol/L	5.13 ± 0.56	5.24 ± 0.61	7.47 ± 2.56	8.02 ± 3.15	< 0.001
HbA1c, %	5.88 ± 0.33	5.87 ± 0.33	7.53 ± 1.32	7.40 ± 1.50	< 0.001
TG, mmol/L	1.70 ± 0.93	1.63 ± 0.92	1.93 ± 1.25	1.77 ± 1.13	< 0.001
TC, mmol/L	4.22 ± 1.05	4.10 ± 0.99	4.24 ± 1.10	4.13 ± 1.08	0.001
HDL-C, mmol/L	1.06 ± 0.30	1.07 ± 0.32	1.02 ± 0.28	1.02 ± 0.29	< 0.001
LDL-C, mmol/L	2.52 ± 0.91	2.40 ± 0.86	2.52 ± 0.91	2.42 ± 0.90	< 0.001
hsCRP, mg/L	2.47 ± 3.02	3.82 ± 4.33	2.96 ± 3.37	4.39 ± 4.70	< 0.001
Creatinine, μmol/L	74.37 ± 14.02	77.41 ± 15.67	74.15 ± 15.47	79.44 ± 19.04	< 0.001
eGFR, mL/min/1.73 m^2^	104.20 ± 20.56	100.70 ± 21.79	103.72 ± 23.08	97.45 ± 23.85	< 0.001
LVEF, %	63.55 ± 6.86	63.18 ± 7.09	62.99 ± 6.87	61.48 ± 8.02	< 0.001
Medications					
DAPT	3558 (97.3)	1113 (97.7)	3008 (98.2)	957 (98.0)	0.105
β-blocker	3234 (88.5)	1007 (88.4)	2827 (92.3)	887 (90.8)	< 0.001
CCB	1734 (47.4)	574 (50.4)	1546 (50.5)	499 (51.1)	0.035
Statins	3534 (96.7)	1097 (96.3)	2932 (95.7)	921 (94.3)	0.005
Nitrate	3580 (97.9)	1122 (98.5)	2997 (97.8)	949 (97.1)	0.184
Coronary procedural data					
LM/three-vessel disease	1449 (39.6)	465 (40.8)	1509 (49.3)	518 (53.0)	< 0.001
Chronic total occlusion	293 (8.0)	95 (8.3)	249 (8.1)	89 (9.1)	0.733
Bifurcation lesions	748 (20.5)	224 (19.7)	601 (19.6)	212 (21.7)	0.503
Moderate to severe calcification	578 (15.8)	205 (18.0)	577 (18.8)	200 (20.5)	0.001
Number of treated vessels	1.41 (0.64)	1.38 (0.63)	1.45 (0.70)	1.52 (0.76)	< 0.001
Number of stents	1.88 ± 1.03	1.87 ± 1.01	1.97 ± 1.09	2.04 ± 1.09	< 0.001
IABP	13 (0.4)	9 (0.8)	46 (1.5)	16 (1.6)	< 0.001
SYNTAX score	11.25 ± 7.32	12.07 ± 7.75	12.24 ± 7.78	13.16 ± 8.22	< 0.001

Values are mean ± standard deviation or n (%)

Abbreviations as in Table 1

Compared with diabetic patients with higher level of NLR, those in other three groups were younger and more likely to be male, with a lower prevalence of hypertension, dyslipidemia, smoking history, previous MI, previous PCI, previous CABG, previous stroke, previous PAD and clinical presentation as ACS. Laboratory test results including neutrophils counts, NLR, FBG, HbA1c, TG, TC, LDL-C, hsCRP and creatinine were significantly higher in the NLR-H/T2DM. However, the level of lymphocyte counts, HDL-C, eGFR and LVEF was relatively lower. Meanwhile, individuals in the other three groups were less likely to have LM/three-vessel disease, moderate to severe calcification and higher SYNTAX score, when compared with those in the NLR-H/T2DM group.

### Relationship between NLR and FBG, HbA1c or hsCRP

Linear regression analysis was performed to evaluate the correlation between NLR and other biomarkers, including FBG, HbA1c and hsCRP (Additional file 1: Table S1). Specifically, NLR was positively correlated with FBG (R = 0.137, P < 0.001) or hsCRP (R = 0.174, P < 0.001) in the whole cohort. In T2DM patients, positive correlation between NLR and FBG (R = 0.157, P < 0.001) or hsCRP (R = 0.161, P < 0.001) were noted. Moreover, there were consistent association of NLR with FBG (R = 0.101, P < 0.001), hsCRP (R = 0.190, P < 0.001) in the non-T2DM cohort. Remarkably, the correlation coefficients between FBG or hsCRP and NLR were relatively weak, which may not be capable of providing sufficient clinical value despite statistically significant correlation.

### Clinical outcomes in different risk groups stratified by NLR and glycemic metabolism status

**Fig. 2:**
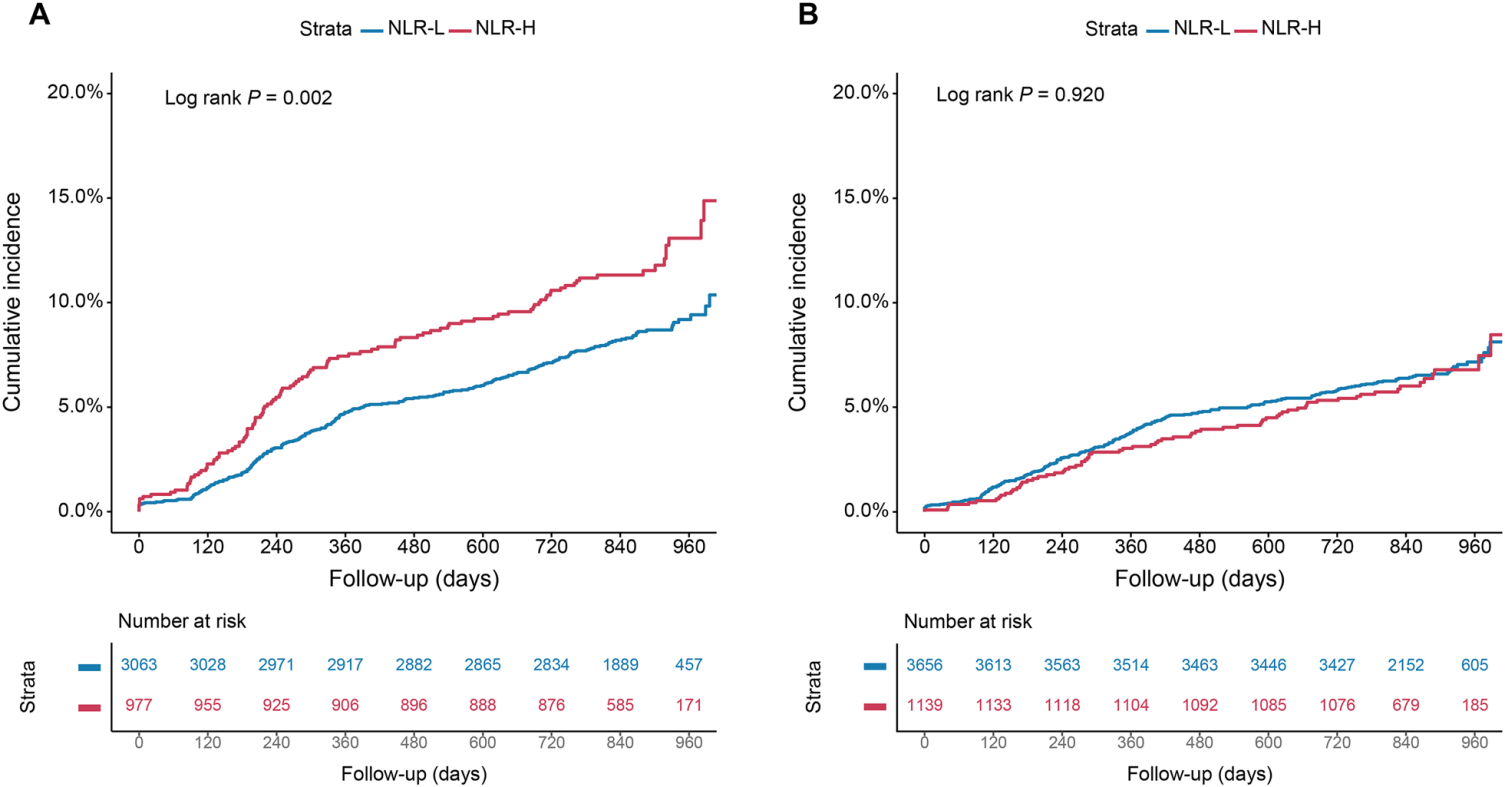
Kaplan–Meier curves for cumulative incidence of MACCEs according to different NLR levels in the T2DM (**A**) and non-T2DM (**B**) groups. Abbreviations as in Table 1

**Fig. 3:**
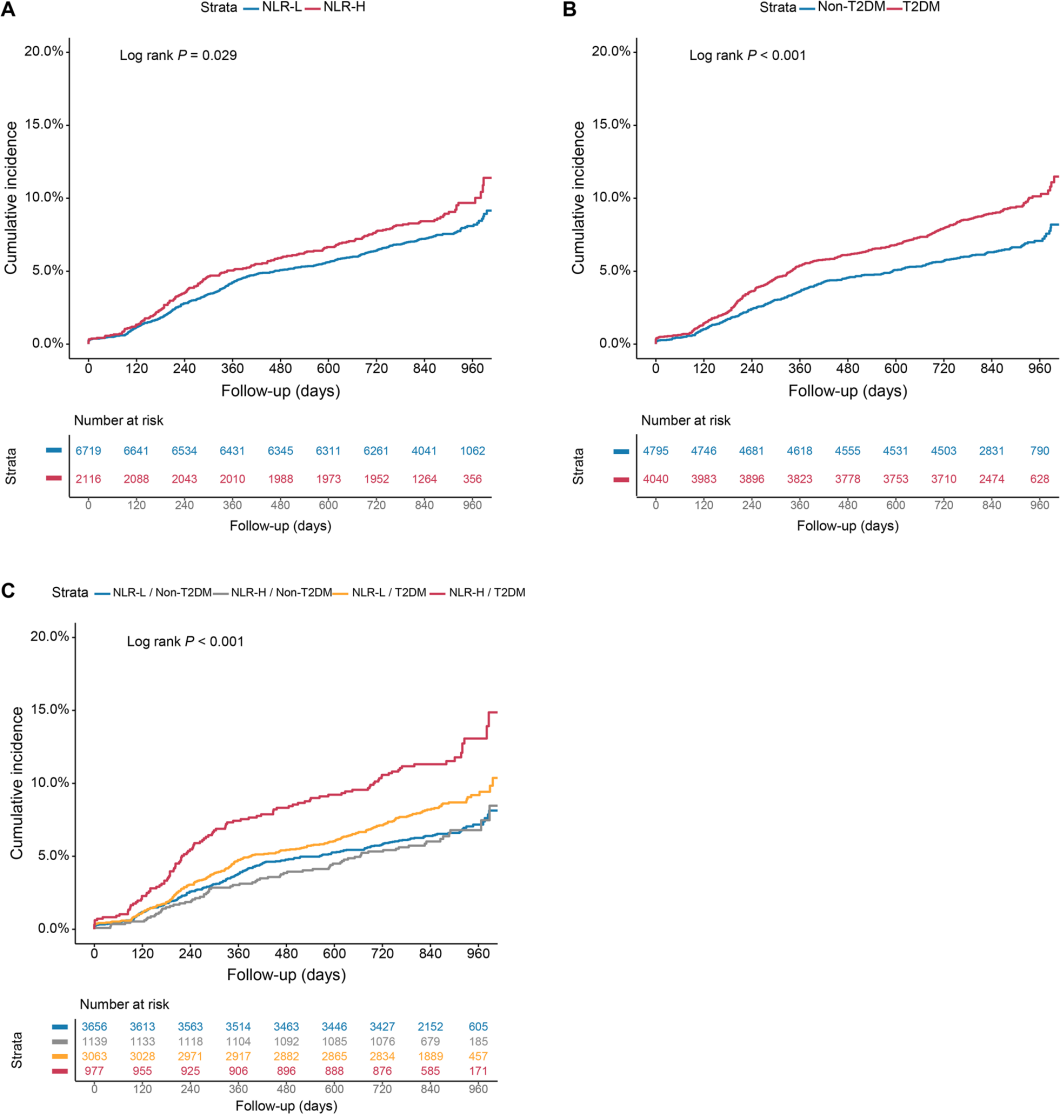
Kaplan–Meier curves for cumulative incidence of MACCE according to different NLR levels (**A**), glycemic metabolism status (**B**), and status of both NLR levels and glycemic metabolism (**C**). Abbreviations as in Table 1

The incidence of MACCE in NLR-H with T2DM or non-T2DM and NLR-L with T2DM or Non-T2DM group was 11.5% (112/977), 6.4% (73/1139), 8.2% (252/3063), 6.5% (237/3656), respectively. As depicted in Figs. 2 and 3 the Kaplan–Meier analysis curves revealed higher risk of MACCEs in patients with NLR-H or T2DM compared with the other groups (log-rank P = 0.026 and P < 0.001, respectively). Additionally, higher level of NLR conferred higher MACCE risk in the T2DM group, with no difference in the non-T2DM group (log-rank P = 0.002 and P = 0.920, respectively)**.** In the adjusted model, there was a significant interaction between glycemic metabolism status and NLR groups after adjustment for confounders (P = 0.046).

In T2DM cohort, a significantly higher risk of MACCEs was observed with higher level of NLR (adjusted HR: 1.36, 95%CI: 1.08 to 1.71, P = 0.009). However, in non-T2DM cohort, no significant different risk of MACCEs was observed at distinct levels of NLR (adjusted HR: 0.89, 95%CI: 0.68 to 1.16, P = 0.378) (Additional file 1: Table S3).

Subsequently, risks of clinical outcomes were compared in four different risk groups by Cox regression model, using the NLR-H/T2DM group as reference (Table 3). In the unadjusted model, patients in the NLR-L/T2DM, NLR-H/non-T2DM and NLR-L/non-T2DM groups were at significantly lower risk of MACCEs (HR: 0.71, 95%CI: 0.57 to 0.89, P = 0.002; HR: 0.55, 95%CI: 0.41 to 0.73, P < 0.001; HR: 0.55, 95%CI, 0.44 to 0.69, P < 0.001; respectively). The results remained statistical significance in multivariable analysis after adjusted for confounding factors (adjusted HR: 0.77, 95%CI: 0.61 to 0.97, P = 0.025; adjusted HR: 0.62, 95%CI: 0.45 to 0.85, P = 0.003; adjusted HR: 0.67, 95%CI, 0.52 to 0.87, P = 0.003; respectively). Collinearity analysis was performed to scrutinize the potential multicollinearity of variables included in the Cox regression model, including age, male sex, hypertension, dyslipidemia, smoking history, previous MI, previous PCI, previous stroke, Previous PAD, ACS, HbA1c, TG, LDL-C, hsCRP, eGFR, LVEF, DAPT, β blocker, LM/three-vessel disease, CTO, moderate to severe calcification, number of treated vessels, number of stents, IABP use and SYNTAX score. As a result, Variance Inflation Factors were all less than 5, indicating that there was no collinearity among the adjusted variables (Additional file 1: Table S2). Consistent results were observed by using the cut-off value as previously reported [24] (Additional file 1: Table S4). Remarkably, there was a significantly lower risk of a composite of all-cause mortality and MI in patients with NLR-L/non-T2DM (adjusted HR: 0.57, 95%CI: 0.35 to 0.93, P = 0.024). Additionally, multivariable Cox proportional hazards regression analysis also revealed the highest risk of MACCEs in patients with NLR-H and T2DM than others (P for trend = 0.009). RCS analysis revealed that there was linear relationship between NLR and the risk of MACCEs irrespective of the univariable and multivariable model (Additional file 1: Figure S1, P for non-linear association > 0.05 for both).

**Table 3:** Predictive value of the NLR level and different glycemic status for MACCEs in univariable and multivariable analysis

Groups	Events/subjects	Univariable		Multivariable	
		HR (95%CI)	*P* value	HR (95%CI)*‡	*P* value†
MACCE					
NLR-H/T2DM	112/977	Reference	NA	Reference	NA
NLR-L/T2DM	252/3063	0.71 (0.57–0.89)	0.002	0.77 (0.61–0.97)	0.025
NLR-H/Non-T2DM	73/1139	0.55 (0.41–0.73)	< 0.001	0.62 (0.45–0.85)	0.003
NLR-L/Non-T2DM	237/3656	0.55 (0.44–0.69)	< 0.001	0.67 (0.52–0.87)	0.003
All-cause mortality and MI					
NLR-H/T2DM	34/977	Reference	NA	Reference	NA
NLR-L/T2DM	59/3063	0.56 (0.36–0.85)	0.006	0.67 (0.43–1.03)	0.070
NLR-H/Non-T2DM	24/1139	0.61 (0.36–1.03)	0.064	0.67 (0.38–1.18)	0.167
NLR-L/Non-T2DM	57/3656	0.45 (0.29–0.69)	< 0.001	0.57 (0.35–0.93)	0.024
All-cause mortality					
NLR-H/T2DM	23/977	Reference	NA	Reference	NA
NLR-L/T2DM	30/3063	0.42 (0.24–0.72)	0.002	0.58 (0.33–1.01)	0.056
NLR-H/Non-T2DM	16/1139	0.60 (0.32–1.14)	0.122	0.62 (0.31–1.26)	0.190
NLR-L/Non-T2DM	35/3656	0.41 (0.24–0.69)	0.001	0.57 (0.31–1.05)	0.072
Myocardial infarction					
NLR-H/T2DM	17/977	Reference	NA	Reference	NA
NLR-L/T2DM	39/3063	0.73 (0.41–1.29)	0.284	0.81 (0.45–1.45)	0.473
NLR-H/Non-T2DM	11/1139	0.56 (0.26–1.19)	0.133	0.69 (0.30–1.58)	0.382
NLR-L/Non-T2DM	30/3656	0.47 (0.26–0.86)	0.014	0.60 (0.30–1.20)	0.149
Stroke					
NLR-H/T2DM	25/977	Reference	NA	Reference	NA
NLR-L/T2DM	49/3063	0.63 (0.39–1.02)	0.061	0.69 (0.42–1.13)	0.140
NLR-H/Non-T2DM	16/1139	0.56 (0.30–1.05)	0.069	0.64 (0.33–1.26)	0.196
NLR-L/Non-T2DM	49/3656	0.53 (0.33–0.86)	0.010	0.67 (0.38–1.16)	0.148
TVR					
NLR-H/T2DM	66/977	Reference	NA	Reference	NA
NLR-L/T2DM	161/3063	0.77 (0.58–1.02)	0.069	0.78 (0.59–1.05)	0.108
NLR-H/Non-T2DM	38/1139	0.48 (0.32–0.72)	< 0.001	0.54 (0.35–0.82)	0.004
NLR-L/Non-T2DM	152/3656	0.60 (0.45–0.81)	< 0.001	0.69 (0.50–0.97)	0.032

^*^Model adjusted for age, male sex, hypertension, dyslipidemia, smoking history, previous MI, previous PCI, previous stroke, Previous PAD, ACS, HbA1c, TG, LDL-C, hsCRP, eGFR, LVEF, DAPT, β blocker, LM/three-vessel disease, CTO, moderate to severe calcification, number of treated vessels, number of stents, IABP use and SYNTAX score

^**†**^P for interaction for the risk of MACCE: LgNLR and glycemic metabolism status (T2DM or Non-T2DM) = 0.172; categorical groups of NLR (low or high) and glycemic metabolism status (T2DM or Non-T2DM) = 0.046

^**‡**^P for trend for the risk of MACCE = 0.009

HR, hazard ratio; CI, confidence interval; NA, not applicable; other abbreviations as in Table 1

### Subgroup analysis

**Fig. 4:**
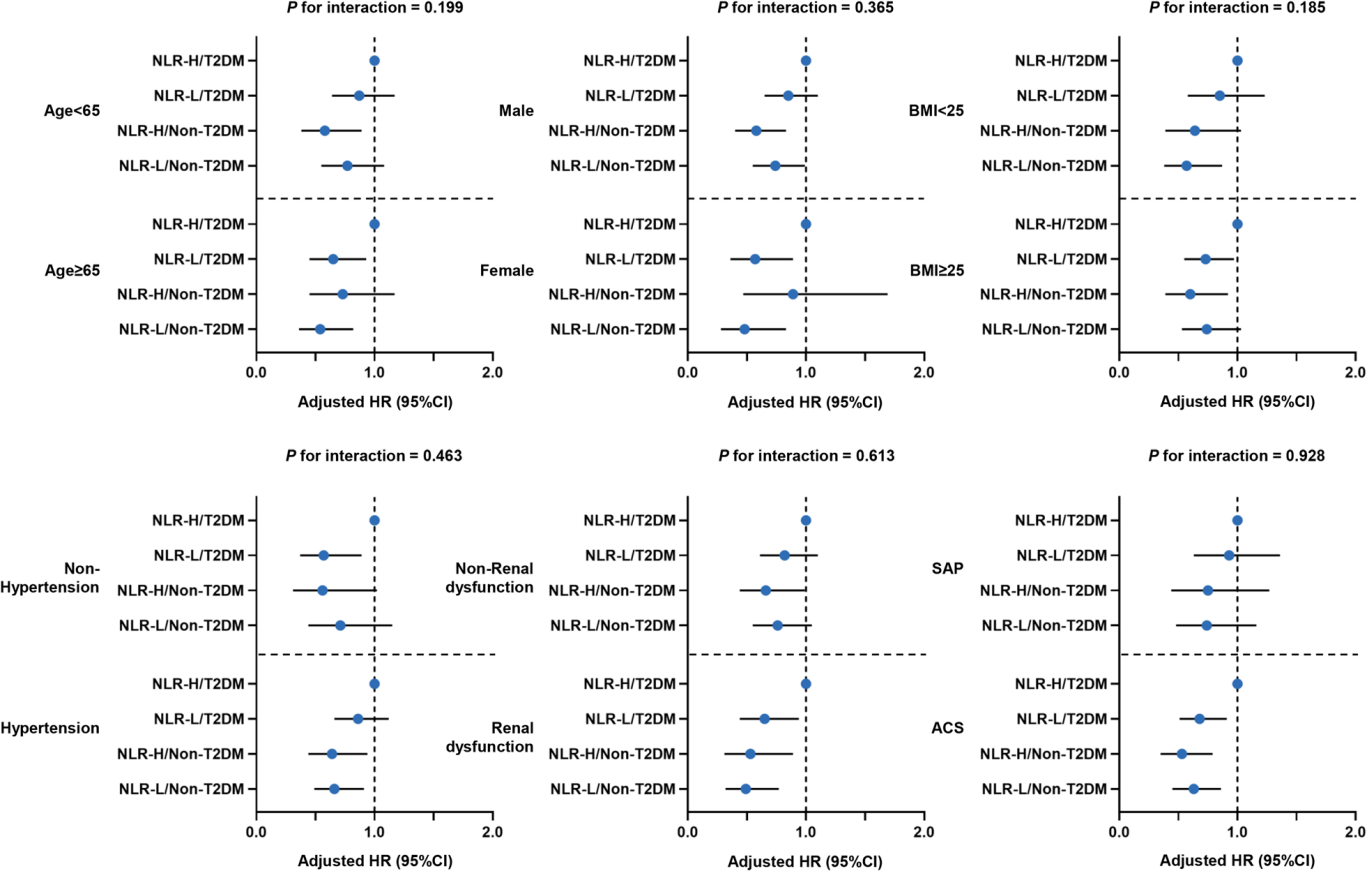
Forest Plot of MACCE According to Various Subgroups. Adjusted for age, male sex, hypertension, dyslipidemia, smoking history, previous MI, previous PCI, previous stroke, Previous PAD, ACS, HbA1c, TG, LDL-C, hsCRP, eGFR, LVEF, DAPT, β blocker, LM/three-vessel disease, CTO, moderate to severe calcification, number of treated vessels, number of stents, IABP use and SYNTAX score. Abbreviations as in Table 1

In subgroup analysis, association between four risk groups and MACCEs were mostly consistent across different subgroups (age, sex, BMI, hypertension, renal dysfunction, and clinical presentation), with indistinguishable interactions (P for interaction > 0.05 for all) (Fig. 4, Additional file 1: Tables S5 and S6).

## Discussion

In this real-world, large-scale, prospective, observational cohort study, the prognosis of diabetic and non-diabetic patients undergoing PCI with different level of NLR was investigated. The major findings of this study are as follows: (1) Higher level of NLR was independently associated with long-term MACCEs in CAD patients with T2DM; (2) T2DM patients with elevated NLR were at significantly increased risks of long-term adverse prognosis compared to non-T2DM ones with lower level of NLR; (3) The association between elevated NLR and adverse clinical events is modified by the glycemic metabolism status in CAD patients after adjustment for confounding factors. Our findings suggested that more precise and accurate risk assessment should be merited in CAD patients with concomitant presence of elevated NLR and T2DM.

### Impact of NLR on prognosis

This study indicated that NLR was significantly correlated with hsCRP in patients with whatever the glycemic status, which is consistent with previous studies [26–28]. Although the role of several specific inflammatory biomarkers (i.e. hsCRP and IL-6) in CAD has been widely investigated [25], NLR is a cheaper, simpler, and more available biomarker of stress and inflammation with satisfactory accuracy. In the CANTOS trial, NLR declined in a dose-dependent manner with canakinumab injection but remained stable in the placebo group, suggesting its potential utility as a therapeutic target to monitor anti-inflammatory responses [29].

In recent years, multiple studies have been conducted to investigate the prognostic value of NLR in various stages of CAD. In patients with CCS, previous studies have demonstrated the predictive value of NLR on cardiac and all-cause mortality after adjustment for confounders [30–32]. Papa et al. revealed that although both absolute neutrophil count and NLR value could predict the risk of cardiac death, neutrophil count lost significance in multivariable model whereas NLR remained significant [30]. As for patients with ACS, previous meta-analyses included large numbers of ACS patients have suggested that a higher NLR on admission was related to an increased risk of major adverse cardiac events and in-hospital and long-term mortality [33, 34], which presumably attribute to extensive early infarction, mechanical complications [35], poor fibrinolytic outcomes, ischemia–reperfusion injury, in-stent restenosis, and suboptimal platelet inhibition [11, 36].

The connection between elevated NLR and adverse prognosis in ACS patients may attributed to several mechanistic pathways. First, increasing evidence suggests that neutrophils act directly to determine myocardial injury [37]. In ACS, neutrophils are functionally activated [38, 39] and local infiltration of these cells has been noted in culprit lesions which indicating their role in mediating destabilization of atherosclerotic plaques [40]. Furthermore, neutrophil-derived microparticles may promote the enhancement of coagulation and elongation of thrombus formation due to its ability of activating platelets and enhancing the expression platelet P-selectin [41]. Second, lymphopenia is induced secondary to increased level of cortisol, catecholamines and proinflammatory cytokines in response to stress, leading to decreased production, tissue redistribution, or lymphocyte apoptosis [42]. Acute lymphopenia, particularly low CD4 + T lymphocytes, is related to poorer clinical outcomes in ST-segment elevated MI patients [43]. Collectively, a high NLR value mirrors two opposite immune pathways and provide better prognostic information than either biomarker alone.

### Joint association of NLR and T2DM with poor prognosis

Previous studies have found that NLR could be used as a predictor of CAD and coronary artery vulnerable plaques in T2DM patients [44]. Coronary artery calcium score detected by computed tomography was also significantly higher in T2DM patients with higher NLR compared to those with lower NLR [45]. In addition, NLR was higher in T2DM patients than in non-T2DM patients [46]. However, the effect of NLR on post-PCI long-term prognosis in CAD patients with or without T2DM still remains uncertain. To date, this study included the largest cohort of CAD patients to estimate the impact of T2DM on NLR and its relationship to poor prognosis.

Notably, significant interaction between NLR and glycemic metabolism status has been found in the present study. Several potential mechanisms may contribute to the interaction effect. First, high NLR values promoted the development and acceleration of diabetic microvascular complications, including diabetic retinopathy, nephropathy, and peripheral neuropathy [46, 47]. Patients with increasing burden of microvascular complications were at risk for higher mortality and poorer cardiovascular outcomes [48]. Second, hyperglycemia with increased NLR was related to increased complexity and severity of coronary lesions [49]. Patients with complicated coronary lesions often presented with additional comorbidities, such as depressed LVEF, and typically were at higher risk of recurrence of adverse cardiac outcomes [50].

In this study, NLR was positively related to FBG in both T2DM and non-T2DM patients. Notably, mean values of HbA1c were only moderately elevated in the present cohort. In line with our findings, Verdoia et al. suggested that there is a significant increase of NLR among patients with T2DM, which was directly correlated with FBG levels, but not with HbA1c [49]. In fact, NLR reflect an acute response, particularly in proinflammatory conditions such as T2DM, and may therefore be associated with transient glycemic indexes rather than variations of long-term glycemic metabolism such as HbA1c.

### Study limitations

There were several limitations need to be addressed. First, selection bias, to some extent, may exist due to missing crucial baseline data in approximately 17.5% of study patients. Second, data regarding dynamic changes in NLR and glycemic metabolism status during follow-up are unavailable. Third, due to the nature of the observational design, potential confounders cannot be adequately controlled. Further well-designed trials are warranted to confirm our results. Fourth, this study was conducted in Chinese patients with CAD undergoing PCI in large-scale tertiary center specializing in cardiovascular diseases [51, 52]. Whether the findings could be generalized to overall populations remains uncertain. Fifth, although this study has the largest cohort in combined status of NLR and T2DM investigations, the cut-off value for risk stratification employed in this work needs to be validated in future studies.

## Conclusions

Presence of T2DM with elevated NLR is associated with worse clinical outcomes in CAD patients undergoing PCI. Categorization of patients with elevated NLR and T2DM could provide valuable information for risk stratification of CAD patients.

## Supplementary Information

**Additional file 1: Table S1.**Correlation analysis between NLR and inflammatory or glycemic indexes in patients with T2DM, without T2DM and whole.** Table S2.**. Univariable and multivariable Cox proportional hazard analysis for primary endpoint.** Table S3**NLR-associated MACCE risk according to different glycemic metabolism.** Table S4.**Predictive value of the NLR level in different glycemic status for MACCEs in univariable and multivariable analysis by using cut-off of 3.04.** Table S5.** Subgroup analysis for the primary endpoint as the unadjusted model.** Table S6.**Subgroup analysis for the primary endpoint as the adjusted model.** Figure S1.**Restricted cubic splines of NLR levels in relation to unadjusted HR(A) and adjusted HR(B) for the risk of MACCE.

## Data Availability

The datasets used and/or analysed during the current study are available from the corresponding author on reasonable request.
