# Supplementary material for: High neutrophil to lymphocyte ratio with type 2 diabetes mellitus predicts poor prognosis in patients undergoing percutaneous coronary intervention: a large-scale cohort study
Source: Cardiovasc Diabetol. 2022 Aug 13;21:156. doi: 10.1186/s12933-022-01583-9 (PMC9375260; doi:10.1186/s12933-022-01583-9)
Supplement: Supplementary file 1 — Additional file 1: Table S1.Correlation analysis between NLR and inflammatory or glycemic indexes in patients with T2DM, without T2DM and whole. Table S2.. Univariable and multivariable Cox proportional hazard analysis for primary endpoint. Table S3NLR-associated MACCE risk according to different glycemic metabolism. Table S4.Predictive value of the NLR level in different glycemic status for MACCEs in univariable and multivariable analysis by using cut-off of 3.04. Table S5. Subgroup analysis for the primary endpoint as the unadjusted model. Table S6.Subgroup analysis for the primary endpoint as the adjusted model. Figure S1.Restricted cubic splines of NLR levels in relation to unadjusted HR(A) and adjusted HR(B) for the risk of MACCE. [file 12933_2022_1583_MOESM1_ESM.docx]

**High neutrophil to lymphocyte ratio with type 2 diabetes mellitus predicts poor prognosis in patients undergoing percutaneous coronary intervention: a large-scale cohort study**

**Additional materials**

| **Table of Contents** | | **Page Number** |
| --- | --- | --- |
| Table S1 | Correlation analysis between NLR and inflammatory or glycemic indexes in patients with T2DM, without T2DM and whole | 2 |
| Table S2 | Univariable and multivariable Cox proportional hazard analysis for primary endpoint. | 3-4 |
| Table S3 | NLR-associated MACCE risk according to different glycemic metabolism | 5 |
| Table S4 | Predictive value of the NLR level in different glycemic status for MACCEs in univariable and multivariable analysis by using cut-off of 3.04 | 6 |
| Table S5 | Subgroup analysis for the primary endpoint as the unadjusted model | 7 |
| Table S6 | Subgroup analysis for the primary endpoint as the adjusted model | 8-9 |
| Figure S1 | Restricted cubic splines of NLR levels in relation to unadjusted HR(A) and adjusted HR(B) for the risk of MACCE. | 10 |

**Table S1 Correlation analysis between NLR and inflammatory or glycemic indexes in patients with T2DM, without T2DM and whole**

| **Variables** | **β** | ***P* value** |
| --- | --- | --- |
| Whole cohort |  |  |
| HbA1c, % | 0.011 | 0.309 |
| FBG, mmol/L | 0.137 | <0.001 |
| hsCRP, mg/L | 0.174 | <0.001 |
| Non-T2DM cohort |  |  |
| HbA1c, % | -0.025 | 0.089 |
| FBG, mmol/L | 0.101 | <0.001 |
| hsCRP, mg/L | 0.190 | <0.001 |
| T2DM cohort |  |  |
| HbA1c, % | -0.024 | 0.125 |
| FBG, mmol/L | 0.157 | <0.001 |
| hsCRP, mg/L | 0.161 | <0.001 |

NLR, neutrophil to lymphocyte ratio; T2DM, type 2 diabetes mellitus; HbA1c, glycosylated [hemoglobin A1c;](http://www.dictall.com/indu/214/213618925C9.htm) FBG, fasting blood glucose; hsCRP, high sensitivity C-reactive protein.

**Table S2 Univariable and Multivariable Cox proportional hazard analysis for primary endpoint.**

| **Variable** | **Univariable analysis** | | | **Multivariable analysis** | | | **VIF** |
| --- | --- | --- | --- | --- | --- | --- | --- |
|  | **HR** | **95% CI** | ***P* value** | **HR** | **95% CI** | ***P* value** |  |
| Category |  |  |  |  |  |  |  |
| NLR-H / T2DM | Reference | - | - | Reference | - | - |  |
| NLR-L / T2DM | 0.99 | 0.76 - 1.28 | 0.919 | 0.77 | 0.61 - 0.97 | 0.025 |  |
| NLR-H / Non-T2DM | 1.28 | 1.07 - 1.53 | 0.007 | 0.62 | 0.45 - 0.85 | 0.003 |  |
| NLR-L / Non-T2DM | 1.82 | 1.45 - 2.27 | <0.001 | 0.67 | 0.52 - 0.87 | 0.003 |  |
| Age, per 1 year | 1.02 | 1.01 - 1.02 | <0.001 | 1.01 | 1.00 - 1.02 | 0.002 | 1.299 |
| Male sex | 1.00 | 0.84 - 1.2 | 0.993 | 1.11 | 0.89 - 1.38 | 0.356 | 1.499 |
| BMI, per 1 kg/m^2^ | 1.00 | 0.98 - 1.02 | 0.965 |  |  |  |  |
| Hypertension | 1.22 | 1.03 - 1.43 | 0.018 | 1.12 | 0.95 - 1.33 | 0.180 | 1.081 |
| Dyslipidemia | 1.08 | 0.92 - 1.27 | 0.368 | 1.03 | 0.87 - 1.21 | 0.768 | 1.055 |
| Smoking history | 0.97 | 0.83 - 1.13 | 0.707 | 0.97 | 0.81 - 1.16 | 0.732 | 1.408 |
| Previous MI | 1.27 | 1.06 - 1.52 | 0.008 | 1.12 | 0.91 - 1.38 | 0.268 | 1.281 |
| Previous PCI | 1.23 | 1.03 - 1.45 | 0.019 | 1.09 | 0.90 - 1.31 | 0.373 | 1.186 |
| Previous CABG | 1.29 | 0.92 - 1.82 | 0.145 |  |  |  |  |
| Previous stroke | 1.48 | 1.19 - 1.83 | <0.001 | 1.32 | 1.06 - 1.64 | 0.014 | 1.051 |
| Previous PAD | 1.08 | 0.82 - 1.43 | 0.597 | 0.92 | 0.69 - 1.23 | 0.579 | 1.038 |
| Clinical presentation as ACS | 1.04 | 0.89 - 1.22 | 0.594 | 1.08 | 0.92 - 1.26 | 0.370 | 1.083 |
| TG, per 1 mmol/L | 0.98 | 0.91 - 1.05 | 0.566 | 1.00 | 0.92 - 1.07 | 0.924 | 1.084 |
| TC, per 1 mmol/L | 1.00 | 0.93 - 1.07 | 0.999 |  |  |  |  |
| HDL-C, per 1 mmol/L | 1.07 | 0.83 - 1.38 | 0.581 |  |  |  |  |
| LDL-C, per 1 mmol/L | 0.99 | 0.91 - 1.08 | 0.868 | 1.01 | 0.92 - 1.10 | 0.874 | 1.052 |
| hsCRP, per 1 mg/L | 1.02 | 1.00 - 1.04 | 0.028 | 1.01 | 0.99 - 1.03 | 0.311 | 1.098 |
| HbA1c, per 1 % |  |  |  | 1.00 | 0.93 - 1.07 | 0.966 | 1.748 |
| Creatinine, per 1 μmol/L | 1.00 | 1.00 - 1.01 | 0.141 |  |  |  |  |
| eGFR, per 1 mL/min/1.73 m^2^ | 1.00 | 0.99 - 1.00 | 0.083 | 1.00 | 1.00 - 1.00 | 0.472 | 1.132 |
| LVEF, per 1 % | 0.98 | 0.97 - 0.99 | <0.001 | 0.99 | 0.98 - 1.00 | 0.095 | 1.168 |
| DAPT | 1.14 | 0.66 - 1.97 | 0.642 | 1.10 | 0.64 - 1.91 | 0.725 | 1.006 |
| β-blocker | 1.32 | 1.00 - 1.75 | 0.052 | 1.28 | 0.96 - 1.70 | 0.089 | 1.022 |
| CCB | 1.14 | 0.98 - 1.33 | 0.09 |  |  |  |  |
| Statins | 0.99 | 0.67 - 1.45 | 0.955 |  |  |  |  |
| Nitrate | 1.11 | 0.64 - 1.93 | 0.705 |  |  |  |  |
| LM/three-vessel disease | 1.45 | 1.25 - 1.69 | <0.001 | 1.22 | 1.03 - 1.45 | 0.022 | 1.301 |
| Chronic total occlusion | 1.33 | 1.04 - 1.70 | 0.024 | 1.19 | 0.91 - 1.54 | 0.199 | 1.090 |
| Bifurcation lesions | 0.87 | 0.71 - 1.05 | 0.152 |  |  |  |  |
| Moderate to severe calcification | 1.28 | 1.07 - 1.54 | 0.008 | 1.12 | 0.93 - 1.36 | 0.245 | 1.059 |
| Number of treated vessels, per 1 vessel | 1.19 | 1.08 - 1.32 | 0.001 | 1.12 | 0.98 – 1.27 | 0.100 | 1.643 |
| Number of stents, per 1 stent | 1.07 | 1.01 - 1.15 | 0.034 | 0.95 | 0.87 - 1.04 | 0.272 | 1.781 |
| IABP | 3.86 | 2.47 - 6.02 | <0.001 | 2.82 | 1.77 - 4.50 | <0.001 | 1.035 |
| SYNTAX score, per 1-point | 1.02 | 1.01 - 1.03 | <0.001 | 1.00 | 0.99 - 1.02 | 0.464 | 1.447 |

HR, hazard ratio; CI, confidence interval; BMI, body mass index; MI, myocardial infarction; PCI, percutaneous coronary intervention; CABG, coronary artery bypass grafting; PAD, peripheral artery disease; ACS, acute coronary syndrome; TG, triglyceride; TC, total cholesterol, HDL-C, high-density lipoprotein cholesterol; LDL-C, low-density lipoprotein cholesterol; eGFR, estimated glomerular filtration rate; LVEF, left ventricular ejection fraction; DAPT, dual antiplatelet therapy; CCB, calcium channel blocker; LM, left main; SYNTAX, synergy between PCI with taxus and cardiac surgery,

Other abbreviations as in table S1.

**Table S3 NLR-associated MACCE risk according to different glycemic metabolism**

| **Groups** | **Events/subjects** | **Univariable** | | **Multivariable** | |
| --- | --- | --- | --- | --- | --- |
|  |  | **HR (95%CI)** | ***P* value** | **HR (95%CI)*** | ***P* value** |
| Whole cohort |  |  |  |  |  |
| LnNLR, per unit | 674/8835 | 1.21 (1.02-1.42) | 0.025 | 1.07 (0.90-1.27) | 0.454 |
| NLR-L | 4899/6719 | Reference | NA | Reference | NA |
| NLR-H | 185/2116 | 1.21 (1.02-1.43) | 0.030 | 1.11 (0.93-1.32) | 0.244 |
| Non-T2DM cohort |  |  |  |  |  |
| LnNLR, per unit | 310/4795 | 1.02 (0.79-1.33) | 0.859 | 0.89 (0.68-1.17) | 0.416 |
| NLR-L | 237/3656 | Reference | NA | Reference | NA |
| NLR-H | 73/1139 | 0.99 (0.76-1.28) | 0.922 | 0.89 (0.68-1.16) | 0.378 |
| T2DM cohort |  |  |  |  |  |
| LnNLR, per unit | 364/4040 | 1.32 (1.08-1.63) | 0.008 | 1.24 (0.99-1.54) | 0.059 |
| NLR-L | 252/3063 | Reference | NA | Reference | NA |
| NLR-H | 112/977 | 1.42 (1.13-1.77) | 0.002 | 1.36 (1.08-1.71) | 0.009 |

*Model adjusted for age, male sex, hypertension, dyslipidemia, smoking history, previous MI, previous PCI, previous stroke, Previous PAD, ACS, HbA1c, TG, LDL-C, hsCRP, eGFR, LVEF, DAPT, β blocker, LM/three-vessel disease, CTO, moderate to severe calcification, number of treated vessels, number of stents, IABP use and SYNTAX score.

HR, hazard ratio; CI, confidence interval; NA, not applicable; other abbreviations as in Table S1.

**Table S4 Predictive value of the NLR level in different glycemic status for MACCEs in univariable and multivariable analysis by using cut-off of 3.04**

| **Groups** | **Events/subjects** | **Univariable** | | **Multivariable** | |
| --- | --- | --- | --- | --- | --- |
|  |  | **HR (95%CI)** | ***P* value** | **HR (95%CI)*‡** | ***P* value†** |
| MACCE |  |  |  |  |  |
| NLR-H/T2DM | 95/804 | Reference | NA | Reference | NA |
| NLR-L/T2DM | 269/3236 | 0.69 (0.55-0.88) | 0.002 | 0.76 (0.60-0.97) | 0.026 |
| NLR-H/Non-T2DM | 58/909 | 0.53 (0.38-0.73) | <0.001 | 0.59 (0.42-0.84) | 0.003 |
| NLR-L/Non-T2DM | 252/3886 | 0.54 (0.42-0.68) | <0.001 | 0.66 (0.50-0.86) | 0.002 |

*Model adjusted for age, male sex, hypertension, dyslipidemia, smoking history, previous MI, previous PCI, previous stroke, Previous PAD, ACS, HbA1c, TG, LDL-C, hsCRP, eGFR, LVEF, DAPT, β blocker, LM/three-vessel disease, CTO, moderate to severe calcification, number of treated vessels, number of stents, IABP use and SYNTAX score.

**†**P for interaction for the risk of MACCE: LgNLR and glycemic metabolism status (T2DM or Non-T2DM) = 0.172; categorical groups of NLR (low or high) and glycemic metabolism status (T2DM or Non-T2DM) = 0.043.

**‡**P for trend for the risk of MACCE = 0.009.

Abbreviations as in Table S1 and Table S3.

**Table S5 Subgroup analysis for the primary endpoint as the unadjusted model**

| **Variables** | **NLR-H/T2DM** | **NLR-L/T2DM** | **NLR-H/Non-T2DM** | **NLR-L/Non-T2DM** | ***P* for interaction** |
| --- | --- | --- | --- | --- | --- |
| **Age** |  |  |  |  | 0.297 |
| <65 | 1.00 (reference) | 0.78 (0.58-1.05) | 0.51 (0.34-0.75) | 0.63 (0.47-0.85) |  |
| ≥65 | 1.00 (reference) | 0.64 (0.45-0.91) | 0.65 (0.42-1.01) | 0.47 (0.33-0.69) |  |
| **Sex** |  |  |  |  | 0.314 |
| Male | 1.00 (reference) | 0.79 (0.61-1.02) | 0.51 (0.36-0.72) | 0.62 (0.48-0.80) |  |
| Female | 1.00 (reference) | 0.52 (0.33-0.80) | 0.73 (0.41-1.30) | 0.38 (0.24-0.61) |  |
| **BMI** |  |  |  |  | 0.179 |
| <25 | 1.00 (reference) | 0.77 (0.53-1.10) | 0.59 (0.38-0.92) | 0.49 (0.34-0.71) |  |
| ≥25 | 1.00 (reference) | 0.68 (0.51-0.90) | 0.51 (0.34-0.76) | 0.60 (0.45-0.79) |  |
| **Hypertension** |  |  |  |  | 0.552 |
| No | 1.00 (reference) | 0.53 (0.35-0.82) | 0.46 (0.26-0.79) | 0.52 (0.34-0.77) |  |
| Yes | 1.00 (reference) | 0.79 (0.61-1.03) | 0.59 (0.42-0.84) | 0.58 (0.44-0.76) |  |
| **Renal dysfunction** |  |  |  |  | 0.658 |
| No | 1.00 (reference) | 0.76 (0.57-1.01) | 0.55 (0.38-0.80) | 0.59 (0.45-0.79) |  |
| Yes | 1.00 (reference) | 0.65 (0.45-0.94) | 0.56 (0.35-0.90) | 0.53 (0.36-0.77) |  |
| **Clinical presentation** |  |  |  |  | 0.758 |
| SAP | 1.00 (reference) | 0.82 (0.56-1.19) | 0.68 (0.42-1.11) | 0.60 (0.41-0.89) |  |
| ACS | 1.00 (reference) | 0.65 (0.49-0.86) | 0.48 (0.33-0.69) | 0.53 (0.40-0.70) |  |

Values are presented with HR (95% CI).

SAP, stable angina pectoris. Other abbreviations as in Table S1 and Table S2.

**Table S6 subgroup analysis for the primary endpoint as the adjusted model**

| **Variables** | **NLR-H/T2DM** | **NLR-L/T2DM** | **NLR-H/Non-T2DM** | **NLR-L/Non-T2DM** | ***P* for interaction** |
| --- | --- | --- | --- | --- | --- |
| **Age** |  |  |  |  | 0.199 |
| <65 | 1.00 (reference) | 0.87 (0.64-1.17) | 0.58 (0.38-0.89) | 0.77 (0.55-1.08) |  |
| ≥65 | 1.00 (reference) | 0.65 (0.45-0.93) | 0.73 (0.45-1.17) | 0.54 (0.36-0.82) |  |
| **Sex** |  |  |  |  | 0.365 |
| Male | 1.00 (reference) | 0.85 (0.65-1.10) | 0.58 (0.40-0.83) | 0.74 (0.55-0.99) |  |
| Female | 1.00 (reference) | 0.57 (0.36-0.89) | 0.89 (0.47-1.69) | 0.48 (0.28-0.83) |  |
| **BMI** |  |  |  |  | 0.185 |
| <25 | 1.00 (reference) | 0.85 (0.58-1.23) | 0.64 (0.39-1.03) | 0.57 (0.38-0.87) |  |
| ≥25 | 1.00 (reference) | 0.73 (0.55-0.97) | 0.60 (0.39-0.92) | 0.74 (0.53-1.03) |  |
| **Hypertension** |  |  |  |  | 0.463 |
| No | 1.00 (reference) | 0.57 (0.37-0.89) | 0.56 (0.31-1.02) | 0.71 (0.44-1.15) |  |
| Yes | 1.00 (reference) | 0.86 (0.66-1.12) | 0.64 (0.44-0.94) | 0.66 (0.49-0.91) |  |
| **Renal dysfunction** |  |  |  |  | 0.613 |
| No | 1.00 (reference) | 0.82 (0.61-1.10) | 0.66 (0.44-0.99) | 0.76 (0.55-1.05) |  |
| Yes | 1.00 (reference) | 0.65 (0.44-0.94) | 0.53 (0.31-0.89) | 0.49 (0.32-0.77) |  |
| **Clinical presentation** |  |  |  |  | 0.928 |
| SAP | 1.00 (reference) | 0.93 (0.63-1.36) | 0.75 (0.44-1.27) | 0.74 (0.48-1.16) |  |
| ACS | 1.00 (reference) | 0.68 (0.51-0.91) | 0.53 (0.35-0.79) | 0.63 (0.45-0.86) |  |

Values are presented with HR (95% CI).

Adjusted for age, male sex, hypertension, dyslipidemia, smoking history, previous MI, previous PCI, previous stroke, Previous PAD, ACS, HbA1c, TG, LDL-C, hsCRP, eGFR, LVEF, DAPT, β blocker, LM/three-vessel disease, CTO, moderate to severe calcification, number of treated vessels, number of stents, IABP use and SYNTAX score.

Abbreviations as in Table S1, Table S2 and Table S5.

**Figure S1 Restricted cubic splines of NLR levels in relation to unadjusted HR(A) and adjusted HR(B) for the risk of MACCE.**


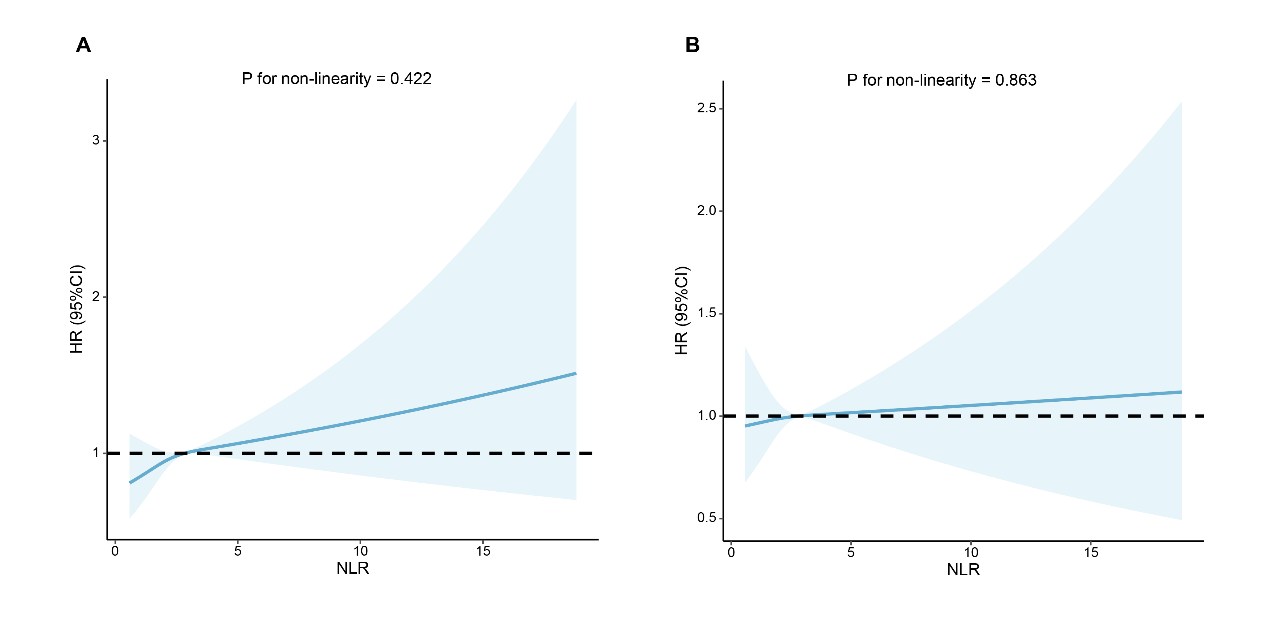


Model adjusted for age, male sex, hypertension, dyslipidemia, smoking history, previous MI, previous PCI, previous stroke, Previous PAD, ACS, HbA1c, TG, LDL-C, hsCRP, eGFR, LVEF, DAPT, β blocker, LM/three-vessel disease, CTO, moderate to severe calcification, number of treated vessels, number of stents, IABP use and SYNTAX score.

Blue line with 95%CI shaded in light blue.

Abbreviations as in Table S1, Table S2 and Table S3.
